# Cataract and glaucoma combined surgery: XEN® gel stent versus nonpenetrating deep sclerectomy, a pilot study

**DOI:** 10.1186/s12886-020-01492-z

**Published:** 2020-06-16

**Authors:** Vincent Theillac, Esther Blumen-Ohana, Jad Akesbi, Pascale Hamard, Alexandre Sellam, Emmanuelle Brasnu, Christophe Baudouin, Antoine Labbe, Jean-Philippe Nordmann

**Affiliations:** 1 Department of Ophthalmology 2, Quinze-Vingts National Ophthalmology Hospital, IHU FOReSIGHT, University Paris Descartes, 28 rue de Charenton, 75012 Paris, France; 2 Department of Ophthalmology 3, Quinze-Vingts National Ophthalmology Hospital, IHU FOReSIGHT, Paris and Versailles Saint-Quentin-en-Yvelines University, Versailles, France; 3 INSERM U968; UPMC Univ Paris 06, UMR_S968, Institut de la Vision; CNRS, UMR 7210; CHNO des Quinze-Vingts, INSERM-DHOS CIC 503, Paris, France

**Keywords:** XEN, Nonpenetrating deep sclerectomy, Primary open angle glaucoma, Microinvasive glaucoma surgery, Cataract, Combined surgery

## Abstract

**Background:**

To compare the efficacy of phacoemulsification (PKE) combined with nonpenetrating deep sclerectomy (NPDS) with mitomycin C (MMC) versus XEN® gel stent with MMC.

**Methods:**

In this nonrandomized, retrospective, comparative, single-center pilot study, 105 consecutive eyes of 75 patients with uncontrolled primary open-angle glaucoma (POAG) and cataract who underwent PKE combined with either XEN implantation (*n* = 47) or NPDS (*n* = 58) between May 2013 and November 2018 were included. The primary outcome was complete success at 9 months, which was defined as intraocular pressure (IOP) ≤18, 15 or 12 mmHg without treatment; qualified success was IOP ≤18, 15 or 12 mmHg with antiglaucoma medications. Secondary outcome measures included the number of antiglaucoma medications, visual acuity (VA), and postoperative adverse events.

**Results:**

Using the 18 mmHg threshold, complete or qualified success was achieved in 69.6 and 89.1% in the PKE + XEN group, and 63.8 and 89.7% in the PKE + NPDS group (*p* = .54 and *p* = .93), respectively, at 9 months. The mean IOP decreased from 20.8 ± 6.8 mmHg to 16.2 ± 2.8 mmHg in the PKE + XEN group (*p* < .001, 18.9% mean drop), and from 21.5 ± 8.9 mmHg to 14.9 ± 3.9 mmHg in the PKE + NPDS group (*p* < .001, 25.6% mean drop). Best-corrected VA significantly improved (*p* < .001) in both groups. The mean number of antiglaucoma medications was significantly reduced from 2.66 ± 1.1 to 0.49 ± 1.0 in the PKE + XEN group (*p* < .001) and from 2.93 ± 0.9 to 0.69 ± 1.2 in the PKE + NPDS group (*p* < .001).

**Conclusions:**

The XEN stent combined with PKE seemed to be as effective and safe as PKE + NPDS at 9 months in this pilot study.

## Background

Glaucoma is one of the world’s leading causes of irreversible blindness and should affect 76 million people worldwide in 2020 [1]. Although trabeculectomy remains the gold standard glaucoma surgery, nonpenetrating deep sclerectomy (NPDS) is an alternative for the surgical treatment of primary open-angle glaucoma (POAG). NPDS has been demonstrated to be effective with fewer surgical complications than trabeculectomy [2]. In the elderly population, cataract is frequently associated with glaucoma when a surgical intervention is needed for glaucoma [3]. Combined phacoemulsification-NPDS (PKE + NPDS) is a safe and effective procedure when cataract is associated with evolutive glaucoma [4]. Its efficacy is comparable to an NPDS performed as a single procedure [5], or a PKE combined with trabeculectomy [6]. Nevertheless, combined PKE + trabeculectomy seemed to have more complications than combined PKE-NPDS [7].

Recently, the development of microinvasive glaucoma surgery (MIGS) with an optimal safety profile promotes combined cataract and glaucoma surgery [8]. Among these techniques, XEN® Gel Stent (Allergan Inc., Irvine, CA, USA), a glutaraldehyde cross-linked porcine collagen tube 6 mm in length and 45 μm in diameter is the only MIGS with *ab interno* implantation [9] that uses the subconjunctival drainage pathway [10]. Cataract surgery combined with XEN has been described and lowered IOP by 25–42% [11, 12]. To the best of our knowledge, no studies have compared PKE + XEN and PKE + NPDS.

The purpose of this study was to compare the efficacy and safety of PKE combined with NPDS (PKE + NPDS) and mitomycin C (MMC) versus PKE combined with the XEN® gel stent (PKE + XEN) and MMC in OAG.

## Methods

This retrospective pilot study was conducted at the Quinze-Vingts National Ophthalmology Hospital, Paris, France, in accordance with the tenets of the Declaration of Helsinki. The medical records of all consecutive patients with no previous glaucoma surgery who underwent PKE + XEN® between June 2017 to November 2018 were reviewed. This group was compared to a matched group (age, sex, surgeon) of patients who underwent PKE + NPDS as first glaucoma surgery between May 2013 and March 2018. Surgical procedures were performed by six glaucoma surgery experts (JA, CB, EBO, EB, PH, and AL).

Preoperative data comprised best-corrected visual acuity (BCVA), subjective refraction, anterior segment and fundus examination, gonioscopy, pachymetry, IOP measurement using a Goldman applanation tonometer, 24–2 ± 10–2 Humphrey visual field (SITA-standard 24–2 and SITA-fast 10–2 programs, with stimulus III-White, of the Humphrey visual field analyzer (Carl Zeiss Meditec, Dublin, CA, USA) with mean deviation (MD), pattern standard deviation (PSD), visual field index (VFI); ganglion cell complex (GCC), and retinal nerve fiber layer (RNFL) optical coherence tomography (OCT Cirrus HD-OCT (Carl Zeiss Meditec, Dublin, CA, USA), and the number of treatments. The BCVA was measured on a Snellen chart and then converted to LogMAR according to the Ferris table [13]. The refractive results were assessed according to the calculation of the change in the spherical component by subtraction of spherical equivalents as previously described [14].

The PKE + XEN® procedure was performed as previously described [11]: topical anesthesia with oxybuprocaine and xylocaine gel, standard phacoemulsification, intraocular injection of a myotic agent, subconjunctival injection of 0.1 mL of 0.2 mg/mL MMC with a 27-G needle beneath the superonasal Tenon and spread away from the limbus, introduction of the preloaded injector through a temporal incision, injector placement in the superonasal quadrant and then 45° rotation towards 12 o’clock when the needle was visible subconjunctivally, injection of the implant ideally through the scleral spur with passage from the sclera to the subconjunctival space 3.0 mm from the limbus, gonioscopic examination to verify the intraocular part of the XEN (about 2 mm) and then visibility and mobility assessment of the subconjunctival part, cautious washing of the viscoelastic, intraocular injection of 0.1 mL of 1 mg/0.1 mL cefuroxime, and hydro-sutures of the corneal edges.

The PKE + NPDS procedure was also performed as previously described [15]: topical anesthesia with oxybuprocaine and xylocaine gel, limbus-based conjunctival flap, application of sponges soaked with 0.2 mg/mL MMC in the sub-Tenon space for 2 min, abundant rinsing and then completion of the first scleral flap until achieving clear cornea, standard PKE, cautious washing of the viscoelastic, intraocular injection of myotic agent, hydro-sutures of the corneal edges, intraocular injection of 0.1 mL of 1 mg/0.1 mL cefuroxime, completion of a deep scleral rectangular flap until careful peeling of the external trabecular membrane up to satisfactory filtration, and conjunctival sutures with resorbable 8/0 Vicryl®.

All patients received topical antibiotics for 1–4 weeks and anti-inflammatory therapy for 6–8 weeks, adjusted according to the situation.

For each patient, the postoperative follow-up consisted of a visit at 1 day, 1 week, and 1, 3, 6, 9 and 12 months after surgery with BCVA, IOP measurement, number of antiglaucoma medications, and notification of a potential complication. The primary outcome was successful surgery at 9 months defined as complete success, corresponding to a postoperative IOP ≤18, 15 or 12 mmHg in the absence of antiglaucoma treatment, and qualified success defined as a postoperative IOP ≤18, 15 or 12 mmHg under one or more antiglaucoma medications; failure was an IOP > 18 mmHg, with or without treatment [16]. Secondary outcomes were IOP decrease, number of antiglaucoma medications, surgery duration, complications, number of needling procedures, visual acuity, refraction, and visit number. We conducted a subgroup analysis in order to observe the effect of postoperative IOP at Day 1 < 9 mmHg [17]. The effective operating times of both techniques were calculated using Hospital Manager Bloc software (Softway Medical®, Meyreuil, France).

Quantitative variables were compared using a Student *t*-test. The chi^2^ test was used for the analysis of qualitative data. The Fisher exact test was used for the analysis of qualitative data when at least one calculated theoretical value was less than 3. Survival curves were generated using the Kaplan-Meier method. All statistical analyses were performed using GraphPad Prism 7® for Windows® (GraphPad Software, La Jolla, CA, USA). Double-sided *p*-values <.05 were considered statistically significant.

## Results

Forty seven eyes of 36 patients and 58 eyes of 39 patients were respectively included in the PKE + XEN® group and in the PKE + NPDS group. There was no difference for age, sex, ethnicity, diabetes, first-degree relatives with POAG, duration of glaucoma, preoperative BVCA, glaucoma type, mean preoperative IOP, and preoperative number of antiglaucoma medications. The mean follow-up period was 8.7 ± 4.2 months for the PKE + XEN group and 20.4 ± 15.6 months for the PKE + NPDS group. The mean preoperative IOP was 20.8 ± 6.8 mmHg in the PKE + XEN group and 21.5 ± 8.9 mmHg in the PKE + NPDS group (*p* = .66). The mean number of preoperative treatments was 2.66 ± 1.1 in the PKE + XEN group and 2.93 ± 0.9 in the PKE + NPDS group. The MD was significantly lower in the PKE + NPDS group than in the PKE + XEN group (− 15.78 ± 9.50 dB versus − 11.54 ± 8.39 dB, *p* = .04) but there was no difference for PSD, VFI, and foveal threshold. The patients’ baseline clinical characteristics are shown in Table 1.

**Table 1:** Baseline characteristics

Characteristic	Total (*n* = 105)	XEN (*n* = 47)	NPDS (*n* = 58)	***p***-value
**Age**, years, mean ± SD		72.1 ± 8.7	69.3 ± 8.2	0.10^a^
**Sex**				
Female	47	21 (45)	26 (45)	0.98^b^
Male	58	26 (55)	32 (55)	
**First-degree relatives with POAG**	27	11 (23)	16 (28)	0.63^b^
**African ethnicity**				
Yes	17	6 (13)	11 (19)	0.39^b^
No	88	41 (87)	47 (81)	
**Diabetes**	21	8 (17)	13 (22)	0.49^b^
**Preoperative Humphrey visual field** (24–2)				
MD [dB], mean ± SD		−11.54 ± 8.39	−15.78 ± 9.50	0.04^a^
PSD [dB], mean ± SD		7.05 ± 3.53	8.00 ± 3.98	0.26^a^
VFI [%], mean ± SD		66.5 ± 29.68	53.31 ± 32.95	0.12^a^
Foveal threshold [dB], mean ± SD		31.11 ± 6.39	28.23 ± 6.35	0.16^a^
**Duration of glaucoma**, years ± SD		12.17 ± 8.81	10.77 ± 8.55	0.62^a^
**Preoperative BCVA**, LogMAR ± SD		0.28 ± 0.16	0.36 ± 0.24	0.15^a^
**Mean pachymetry**, μm ± SD		521.8 ± 31.4	518 ± 47.21	0.67^a^
**Cup-to-disc ratio** ± SD		0.80 ± 0.18	0.85 ± 0.16	0.21^a^
**Disease type**				
POAG Pigment dispersion PEXG NTG High myopia Cortisone-induced	77 (73) 3 (3) 16 (15) 1 (1) 3 (3) 5 (5)	34 (73) 2 (4) 8 (17) 1 (2) 0 (0) 2 (4)	43 (74) 1 (2) 8 (14) 0 (0) 3 (5) 3 (5)	0.71^b^
**Antiglaucoma medication**, mean ± SD		2.66 ± 1.07	2.93 ± 0.88	0.16^a^
**Preoperative SLT**		14 (30)	28 (48)	0.1^b^
**Preoperative acetazolamide tablet**, mean ± SD		0.86 ± 1.24	0.66 ± 1.04	0.38^a^

The results are displayed as *n* (%) for categorical variables

*SD* standard deviation, *NPDS* nonpenetrating deep sclerectomy, *HFA* Humphrey visual field analyzer, *MD* mean deviation, *PSD* pattern standard deviation, *VFI* visual field index, *BCVA* best corrected visual acuity, *POAG* primary open-angle glaucoma, *PEXG* pseudoexfoliative glaucoma, *NTG* normal-tension glaucoma, *SLT* selective laser trabeculoplasty

^a^ Student *t*-test

^b^ chi-squared test

Complete and qualified success at 9 months, using 18 mmHg threshold, was achieved, respectively, in 69.6 and 89.1% in the PKE + XEN group and 63.8 and 89.7% in the PKE + NPDS group (*p* = .54 and *p* = .93) (Table 2).

**Table 2:** Combined surgery success at 9 months

	XEN (*n* = 46 eyes)	NPDS (*n* = 58 eyes)	***p***-value
**Complete success***[without* treatment]			
≤ 18 mmHg	32 (69.6)	37 (63.8)	0.54
≤ 15 mmHg	25 (54.4)	29 (50.0)	0.67
≤ 12 mmHg	13 (28.3)	17 (29.3)	0.91
**Qualified success **[*with* treatment]			
≤ 18 mmHg	41 (89.1)	52 (89.7)	0.93
≤ 15 mmHg	32 (69.6)	38 (65.5)	0.66
≤ 12 mmHg	18 (39.1)	21 (36.2)	0.76
**Failure**	5 (10.9)	6 (10.3)	0.93

The results are displayed as *n* (%)

**Fig. 1:**
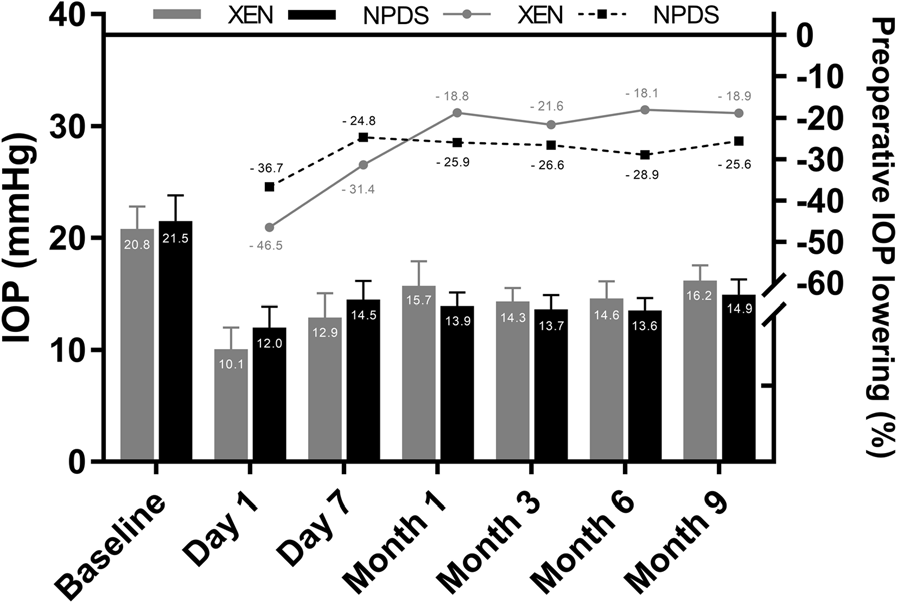
Mean intraocular pressure (IOP) and mean IOP lowering from baseline through 9 months of follow-up, by surgery group. Error bars represent 95% confidence intervals

**Fig. 2:**
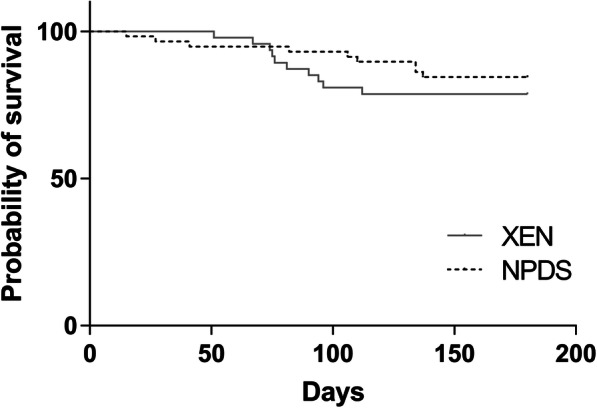
Kaplan-Meier survival analyses of the probability of avoiding glaucoma medication after surgery

The mean IOP at 9 months was 16.2 ± 2.8 in the PKE + XEN group (*p* < .001 compared to preoperative IOP) and 14.9 ± 3.9 mmHg in the PKE + NPDS group (*p* < .001 compared to preoperative IOP). There was no mean IOP difference between the two groups at 9 months (*p* = .25). The mean IOP lowering between the preoperative and the 9-month evaluation was − 18.9 ± 5.2% in the PKE + XEN group and − 25.6 ± 4.3% in the PKE + NPDS group (*p* = .39) (Fig. 1). The mean number of preoperative treatments decreased from 2.66 ± 1.1 to 0.49 ± 1.0 in the PKE + XEN group (*p* < .001) and from 2.93 ± 0.9 to 0.69 ± 1.2 in the PKE + NPDS group at 9 months (*p* < .001). The treatment-free survival curves (Fig. 2) did not differ significantly between the two groups (*p* = .39).

BCVA was significantly improved by surgery (*p* < .001 in both groups) with no postoperative difference between the two groups (Table 3).

**Table 3:** Postoperative visual acuity outcome

	XEN (*n* = 45 eyes)	NPDS (*n* = 53 eyes)	***p***-value
**Postoperative VA**, LogMAR mean ± SD	0.07 ± 0.13	0.12 ± 0.17	0.12
**VA evolution**, LogMAR ± SD	−0.21 ± 0.17	−0.24 ± 0.31	0.65

*VA* visual acuity, *NPDS* nonpenetrating deep sclerectomy, *MAR* minimal angle of resolution, *SD* standard deviation

The preoperative and final postoperative spherical equivalents were similar in both groups (Table 4).

**Table 4:** Comparison of spherical equivalent evolution in PKE + XEN and PKE + NPDS

	XEN (*n* = 45 eyes)	NPDS (*n* = 53 eyes)	***p***-value
**Preoperative spherical equivalent**, mean ± SD	−1.23 ± 4.33	−1.22 ± 3.33	0.99
**Postoperative spherical equivalent**, mean ± SD	− 0.55 ± 1.03	− 0.55 ± 1.16	0.99
**Spherical component variation**, mean ± SD	0.67 ± 4.18	0.71 ± 3.18	0.97

*NPDS* nonpenetrating deep sclerectomy, *SD* standard deviation

The mean operating time was 52.97 ± 14.37 min in the PKE + NPDS group versus 39.09 ± 12.75 min in the PKE + XEN group (*p* < .001). As detailed in Table 5, the number of immediate complications was similar in both groups. No cases of loss of light perception, nor IOP < 5 mmHg leading to hypotony related maculopathy were recorded.

**Table 5:** Postoperative complications in XEN and NPDS eyes

Complication	XEN (*n* = 47 eyes)	NPDS (*n* = 58 eyes)	***p***-value
**Choroidal detachment**	0	1 (1.7)	0.91
**Leak/dehiscence**	1 (2.1)	2 (3.5)	0.69
**Conjunctival suture**	0	2 (3.5)	0.20
**Goniopuncture**		24 (41.4)	
**2nd surgery**	2 (4.3)	2 (3.5)	0.82
**NPDS**	2 (4.3)	0	
**Trabeculectomy**	0	1 (1.7)	
**Trans-scleral cyclodiode laser**	0	1 (1.7)	
**Use of aqueous suppressant for suspected bleb encapsulation**	7 (14.9)		
**Iris incarceration**	1 (2.1)	3 (5.2)	0.42
**Bleb revision surgery**	1 (2.1)	1 (1.7)	0.88

The results are displayed as *n* (%)

**Fig. 3:**
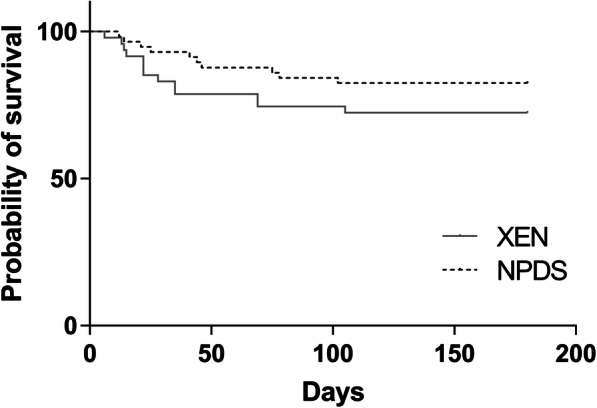
Kaplan-Meier survival analyses of the probability of avoiding needling after surgery

There was no significant difference in the number of needling procedures between the two groups: 14 patients required ≥1 needlings (30%) in the PKE + XEN group and 11 (19%) in the PKE + NPDS group (*p* = .19). The survival curves (Fig. 3) of the number of needling procedures did not differ between the two groups (*p* = .17). Twenty-four goniopunctures (41.4% of the patients) were performed in the PKE + NPDS group, with a mean completion time of 90.7 ± 31.9 days.

IOP < 9 mmHg on Day 1 after PKE + NPDS surgery was associated with complete success (*p* = .02). This was not the case in the PKE + XEN group (*p* = .37).

## Discussion

In POAG patients, cataract is frequently associated with glaucoma when a glaucoma surgical intervention is needed. The development of microinvasive glaucoma surgery (MIGS) with an optimal safety profile and rapid recovery has recently renewed interest in combined cataract and glaucoma surgery. In the present study, there was no statistically significant difference in success rate between PKE combined with XEN or PKE combined with NPDS after a 9-month follow-up period.

The NPDS procedure supports the goal of a safer profile than trabeculectomy [2], as does MIGS. Despite surgical difficulties and the need for surgical experience, NPDS is a common procedure in France: it accounted for 64.7% of the number of trabeculectomies in 2014 [18]. Eldaly et al. [2] indicated that success in deep sclerectomy did not differ from that in trabeculectomy.

The IOP results of combined PKE + XEN were consistent with those described in the literature [11, 12]. Complete and qualified success rates ranged, respectively, from 50% [19] to 90% [20] and from 50% [19] to 97.5% [21], in accordance with our results.

Publications on combined PKE + NPDS surgery have reported IOP results consistent with our findings [22]. Previous studies have found an IOP-lowering effect around 38% [22] for combined PKE + NPDS surgery. Our results were more modest with IOP lowered by 25.6%. This could be explained by the especially low preoperative IOP in the PKE + NPDS group (21.5 mmHg) compared to the preoperative IOP reported in the literature [22].

Day 1 IOP appears to be a significant prognostic factor in combined PKE + NPDS surgery, as shown by Garcia-Pérez et al. Day 1 IOP < 9 mmHg was associated with a higher success rate and fewer treatments and goniopunctures [17]. In the present study we had similar results with a complete success rate significantly higher in the subgroup of patients undergoing PKE + NPDS with an IOP < 9 mmHg at Day 1 (*p* = .02). However, this was not observed for the PKE + XEN procedure in our study. In NPDS, IOP at Day 1 is an indicator of adequate inner scleral flap dissection and juxtacanalicular trabecular meshwork removal [17], which provides a better result. Although XEN implant provides constant resistance with a steady-state pressure of approximately 6–8 mmHg following an uncomplicated implantation under normal physiologic conditions and without conjunctival resistance to the outflow [9], Day 1 IOP does not seem to be a predictor of long-term outcomes for the XEN, whether it was a standalone or a combined procedure [23].

Combined PKE + trabeculectomy remains the gold standard surgical treatment for cataract and glaucoma surgery [24], but it has potentially severe surgical complications [7]. In the present study, we found no major complications in both groups during the 9-month study period. The short follow-up duration and the small number of patients who underwent PKE + XEN did not allow concluding on long-term complications such as extrusion, avascular bleb, blebitis, or endophthalmitis [25, 26]. NPDS postoperative management allows fine adjustments with goniopuncture, although XEN enables only bleb management with needling and surgical revision. The needling rate in the PKE + XEN group was within the low range of those found in the XEN literature (between 30.7% [27] and 47% [16]). The needling rates were not significantly different between the two groups while one surgical revision in each group was observed among the patients included in this pilot study. Seven patients (14.9%) had a use of aqueous suppressant for suspected bleb encapsulation with Dorzolamide 2%-Timolol 0.5% twice a day during 2 weeks as a postoperative management. This particular treatment has been previously recommended by an expert review as a first step treatment if a bleb encapsulation development was suspected while needling was suggested if aqueous suppressants failed [28]. This procedure might explain the low needling rate in our study.

Patients who underwent combined PKE + NPDS surgery had more severe MD than those who underwent PKE + XEN. Nevertheless, no significant difference in the other parameters of glaucoma severity was found in this study (PSD, visual field index, C/D). Patients referred for their first glaucoma surgery in Europe had a mean MD of − 13.8 dB, consistent with that found in our two groups [29]. The difference in MD could be explained by the surgeon’s caution regarding the new PKE + XEN technique and its unfamiliar outcomes, which led to the selection of patients with a less impaired visual field to prevent the potential risk of sudden visual loss (“wipe-out”).

There is, to date, no study assessing the cost of XEN [30]. The economic rationale proposed is based on combined surgery: the rapid implantation of XEN would save operating time with regard to filtering surgery compared to conventional filtering surgery. This time savings, which could be used to perform other surgical procedures, would offset the extra cost related to the XEN implant. In the present study, there was indeed a clinically significant 14.82-min difference in surgery duration, which could make it possible to perform more procedures if XEN is used. This finding needs to be confirmed in a complete operating program and at least on a monthly operating volume, but XEN shows a fast learning curve by both experienced surgeons and novice residents [31], unlike the NPDS, which implies delicate dissection. However, the quality-of-life indicators remain to be determined.

We acknowledge limitations to this study. First, we conducted a retrospective study, and the conclusions would be strengthened by a randomized controlled trial. However, in the PKE + XEN group, patients were consecutively and exhaustively included. The PKE + XEN group was matched with PKE + NPDS on age, sex, and surgeon. Although, for a limited number of patients, we included both eyes in the statistical analysis, the large number of subjects included in the present study might have limited this bias. In our pilot study, the mean follow-up was of only 8.7 ± 4.2 months so it was not possible to conclude on the long-term efficacy of combined PKE + XEN surgery. XEN must necessarily be assessed in the long term: it has been developed to reduce the risk of hypotony without considering conjunctival resistance [32], but the resistance of XEN to aqueous humor flow is increased by that of conjunctival healing [9]: the long-term efficacy of XEN could only be assessed with mature blebs [32].

## Conclusions

In this pilot study, the XEN implant combined with PKE confirmed a favorable risk/benefit ratio, comparable to that of PKE combined with NPDS. This new combined technique is safe and effective, and significantly lowers IOP and the number of anti-glaucoma treatments in the medium term. The operating time of PKE + XEN surgery is significantly shorter than that of PKE + NPDS surgery.

Thus, combined PKE + XEN surgery appears to be a promising procedure, although its place in the surgical arsenal of glaucoma treatment remains to be clarified.

## Data Availability

The datasets used and/or analyzed during the current study are available from the corresponding author on reasonable request.

## Abbreviations

PKE: Phacoemulsification
NPDS: Nonpenetrating deep sclerectomy
MMC: Mitomycin C
POAG: Primary open-angle glaucoma
IOP: Intraocular pressure
VA: Visual acuity
MIGS: Minimally/Micro-invasive glaucoma surgery
BCVA: Best-corrected visual acuity
MD: Mean deviation
PSD: Pattern standard deviation
VFI: Visual field index
GCC: Ganglion cell complex
RNFL: Retinal nerve fiber layer
